# Cerebral manifestations of falciparum malaria in adults: more than meets the eye

**DOI:** 10.1016/j.pt.2025.02.005

**Published:** 2025-03-10

**Authors:** Samuel C. Wassmer, Sanjib Mohanty, Praveen K. Sahu, Angelika Hoffmann

**Affiliations:** 1 Department of Infection Biology, Faculty of Infectious and Tropical Diseases, London School of Hygiene & Tropical Medicine, London, UK; 2 Department of Molecular Biology and Infectious Diseases, Community Welfare Society Hospital, Rourkela, Odisha, India; 3 Institute of Diagnostic and Interventional Neuroradiology, University Hospital Bern, University of Bern, Bern, Switzerland

## Abstract

The application of neuroimaging techniques to patients with *Plasmodium falciparum* infection has uncovered a wide range of brain changes not only in cerebral malaria but also in noncomatose patients. We propose several hypotheses to unify findings across the spectrum of clinical malaria in adults and highlight the urgent need to evaluate potential long-term effects of cerebral alterations on neurocognition in this understudied age group.

## Falciparum malaria in adults

*Plasmodium falciparum* is a major global infection, accounting for most of the 597 000 malaria-related deaths in 2023 [1]. It results in a wide spectrum of disease that varies with age and transmission intensity, and it ranges from asymptomatic infection to multiorgan failure and death [2]. The pathophysiology of severe malaria is complex, involving a combination of intravascular **sequestration** (see Glossary) of *P. falciparum*–infected red blood cells (iRBCs), resulting tissue hypoxia, exaggerated inflammatory responses, and coagulopathy [3]. Multiple and overlapping clinical syndromes often occur in adult patients with severe malaria, including acidosis, severe malaria anemia, jaundice, acute kidney injury (AKI), and **cerebral malaria** (CM) [4]. Severe malaria develops in individuals with little to no immunity. It affects predominantly children under 5 years of age in areas of high stable malaria transmission, as their immunity builds up with regular exposure to the malaria parasite, and in older children and adults residing in low and unstable transmission areas, as their acquired immunity remains limited over time [5]. In the latter setting, severity and mortality rates increase with age, likely because of a greater incidence of multiorgan dysfunction in adults [5,6].

CM is a severe and life-threatening complication of falciparum malaria defined by the World Health Organization as an acute encephalopathy characterized by a coma or Glasgow Coma Scale (GCS) score <11 [4], with a mortality rate up to 25% [4,7]. A wide range of persistent neurocognitive sequelae with life-altering functional consequences occurs in 25% of pediatric survivors [8,9]. Mechanisms underlying the development of CM are multifarious [10], and several studies have recently suggested distant organ effects on the brain caused by concomitant complications during severe malaria. These include AKI, which can lead to increased blood–brain barrier (BBB) permeability, especially in the hippocampus, and overall deranged physiological brain functions [11], but also liver dysfunction through increased circulating ammonia, free heme, hemozoin, and reactive oxygen species [12]. The host gut microbiome has been postulated to influence the development of CM, further complicating its pathogenesis picture [13].

Although CM is commonest in children in Africa, it also occurs in adults living in low or seasonal transmission settings, and the pathogenic mechanisms and long-term consequences of CM in adults remain vastly underexplored [6,11]. Studies using serial magnetic resonance imaging (MRI) of the brain have recently challenged the current understanding of CM in adults, highlighting previously unknown cerebral changes during falciparum malaria, regardless of the presence of coma [14]. These novel findings across the falciparum malaria clinical spectrum are discussed below and suggest potential long-lasting clinical consequences, which have never been rigorously assessed in adults.

## Neuroimaging concepts and findings in CM

**Figure 1.**
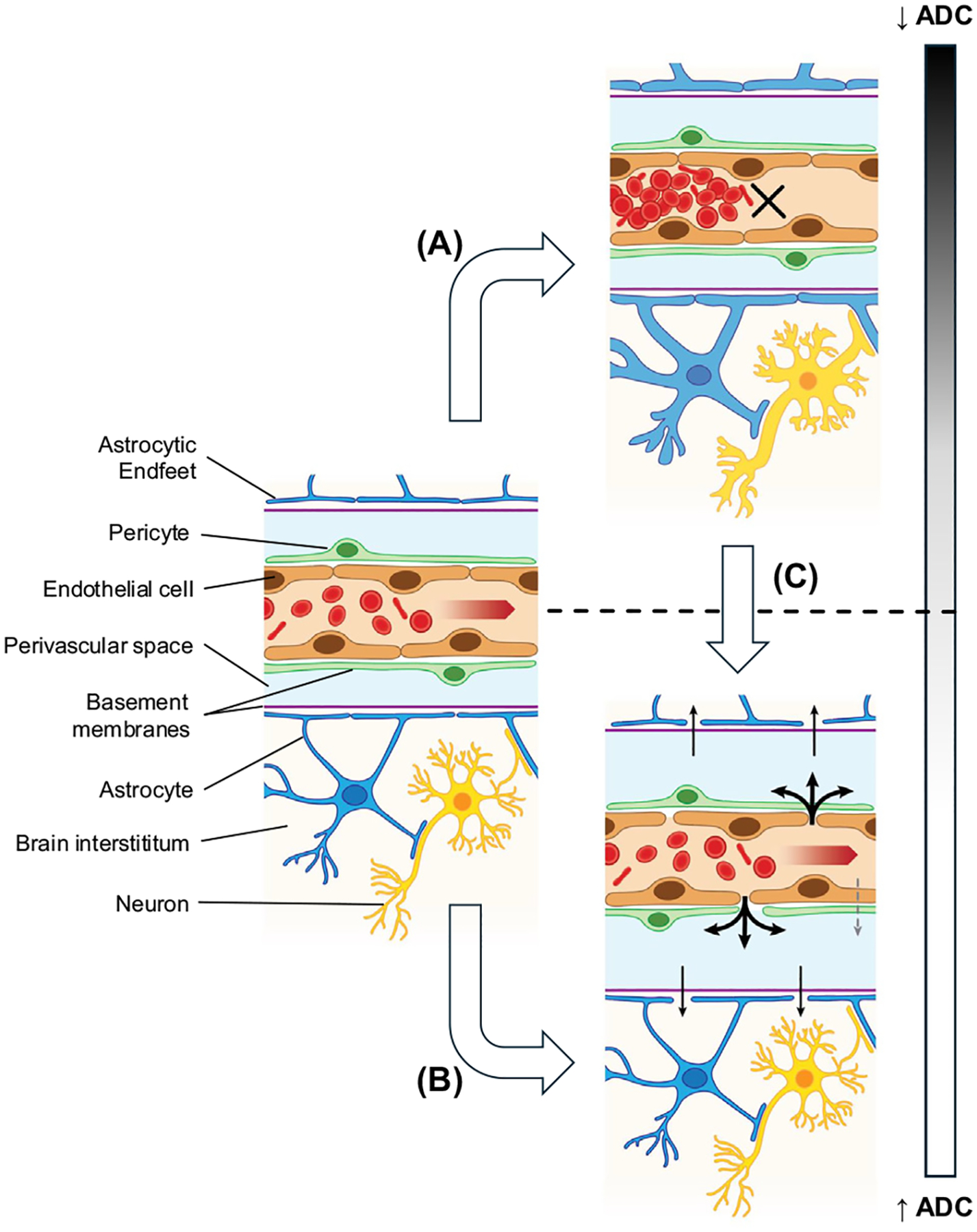
Pathogenic mechanisms associated with different types of edemas and their diffusion-weighted imaging signatures. The neurovascular unit (NVU) is composed of brain endothelial cells, pericytes, basement membranes, astrocytes and their endfeet and neurons, and forms the interface between the blood–brain barrier (BBB) and brain parenchyma. In physiological conditions, it guarantees brain homeostasis and facilitates nutrient delivery and metabolic waste clearance. The NVU reacts to pathological conditions that can be measured by diffusion-weighted imaging and its quantitative measure, the apparent diffusion coefficient (ADC). In case of disrupted blood flow, oxygen delivery is insufficient and cells of the NVU swell, especially astrocytes, leading to cytotoxic edema (A). Cytotoxic edema has low (dark) values on ADC maps and indicated reduced Brownian molecular movement in the narrowed extracellular space. If the BBB disrupts and fluid reaches the extracellular and parenchymal space, vasogenic edema occurs (B). This increase in fluid appears bright on ADC maps and is characterized by high ADC values with increased Brownian molecular movement compared with physiological conditions. In hypoxic conditions, cytotoxic edema precedes vasogenic edema (C). BBB disrupts via paracellular (black arrows) and potentially transcellular pathways (dashed gray arrow).

A study conducted in Malawi demonstrated for the first time that fatal pediatric CM is associated with severe brain swelling and consecutive brainstem herniation, ultimately leading to respiratory arrest [15]. Remarkably, prior neuroimaging studies in adult patients with CM only showed mild to moderate brain swelling [16,17], suggesting an alternative cause of death in this age group. The posterior, parietooccipital areas of the brain were most affected, and the swelling reversed after effective antiparasitic treatment in nonfatal cases [18]. In fatal cases, serial brain MRI in adults showed that global brain hypoxia, assessed by diffusion-weighted imaging (DWI), was more pronounced than in nonfatal cases [19]. DWI is an MRI sequence that allows the detection of hypoxia by visualizing cytotoxic edema within minutes of cell swelling. The latter occurs in cells of the neurovascular unit as a result of insufficient oxygen supply. Cytotoxic edema leads to a narrowing of the extracellular space and thus to a reduction of Brownian molecular motion in that compartment. This phenomenon can be measured with DWI, was first described in 1965 [20], and can be quantitatively measured by **apparent diffusion coefficient (ADC)** maps derived from DWI. Cytotoxic edema does not cause swelling but can be considered as the initial step leading to vasogenic edema and subsequent brain volume increase [21]. Cytotoxic edema triggers BBB disruption with an increase of fluid seepage in the extracellular space after vessels become more permeable. Both cytotoxic and vasogenic edema can be visualized on ADC maps. A decrease in ADC maps signals cytotoxic edema, with a reduction of Brownian molecular motion in the extracellular space compared with physiological condition. Conversely, an ADC increase indicates vasogenic edema, associated with a rise in Brownian molecular motion compared with baseline (Figure 1).

During CM, the high parasite biomass of sequestered iRBCs in the brain results in a mechanical obstruction of the microcirculation, leading to local blood flow reduction, a decrease in oxygen supply, and consecutive hypoxia. In adults with nonfatal CM, an ADC decrease occurs predominantly in the basal ganglia and subcortical white matter during the acute phase of the disease but also in the corpus callosum and hippocampus [19,22–24]. These brain areas are the first affected by hypoxia during CM, and ADC alterations can reverse rapidly upon prompt treatment [19,23]. However, ADC does not always normalize following therapy and can persist if the antimalarial treatment was delayed or the initial cerebral damage pronounced [19,23]. ADC values can also increase post-treatment in the subacute disease stage. It can take weeks to months for ADC to normalize in prolonged CM cases with persistent brain injury such as basal ganglia infarction and microhemorrhages [23]. The latter injuries, detected using susceptibility-weighted imaging, occur predominantly in the subcortical white matter, corpus callosum, internal capsule, and basal ganglia and increase over time [25].

Similar microhemorrhage distribution patterns have been reported in other severe diseases with reduced oxygen supply to the brain, including coronavirus disease (COVID-19), cardiac arrest, high-altitude cerebral edema, severe respiratory failure, and disseminated intravascular coagulation [26–30]. These similitudes highlight that specific brain areas are more susceptible to hypoxia, regardless of the hypoxic cause. In contrast to the above-mentioned conditions, except for high-altitude cerebral edema, hypoxia in CM can rapidly reverse once antimalarial treatment is administered and sequestered iRBCs are cleared from the microcirculation, restoring blood flow. It is noteworthy that the macrocirculation can also be affected in adult CM, leading to reversible vasoconstrictions [31]. Little is known about macrovascular alterations in CM, which warrants further investigations.

## Neuroimaging findings in noncerebral malaria

Recent neuroimaging studies have demonstrated that brain pathological alterations identified by MRI are not limited to patients with CM but are also seen in patients with **uncomplicated malaria (UM)** and patients with severe non-CM, questioning the definition of ‘cerebral’ malaria in adults. Cytotoxic lesions of the corpus callosum have been reported in patients with UM [24,32]. This type of lesion occurs in many conditions, including drug therapy, other infections, trauma, or metabolic disorders. The exact underlying mechanism is unknown, but increased levels of cytokines and extracellular glutamate have been associated with cytotoxic lesions in the corpus callosum. The use of ADC maps has also allowed the identification of subtle changes in these patients, including a high frequency of ADC increase [19], indicative of mild vasogenic edema [33]. Mild brain swelling was also described in up to 50% of patients with severe non-CM [16,34]. Additionally, visual ADC decrease in the basal ganglia, in line with cytotoxic edema, was evident in 13%–19% of severe non-CM cases [16,34], demonstrating that hypoxic brain injury can also occur in the absence of coma. This was exacerbated in patients with severe kidney involvement, indicating that the degree of brain hypoxia is linked to kidney injury [34]. ADC increase was also noted in some of these patients, with less pronounced but measurable brain involvement. These neuroimaging findings reflect the wide spectrum of severe non-CM disease in adults, with MRI patterns ranging from decreased ADC in the basal ganglia and subcortical white matter seen in CM to increased ADC reported in UM. It is likely that several factors contribute to brain involvement, and the severity of brain injury may depend on the degree of other organ involvement [34].

## Proposed sequence of pathogenic mechanisms

### CM

**Figure 2.**
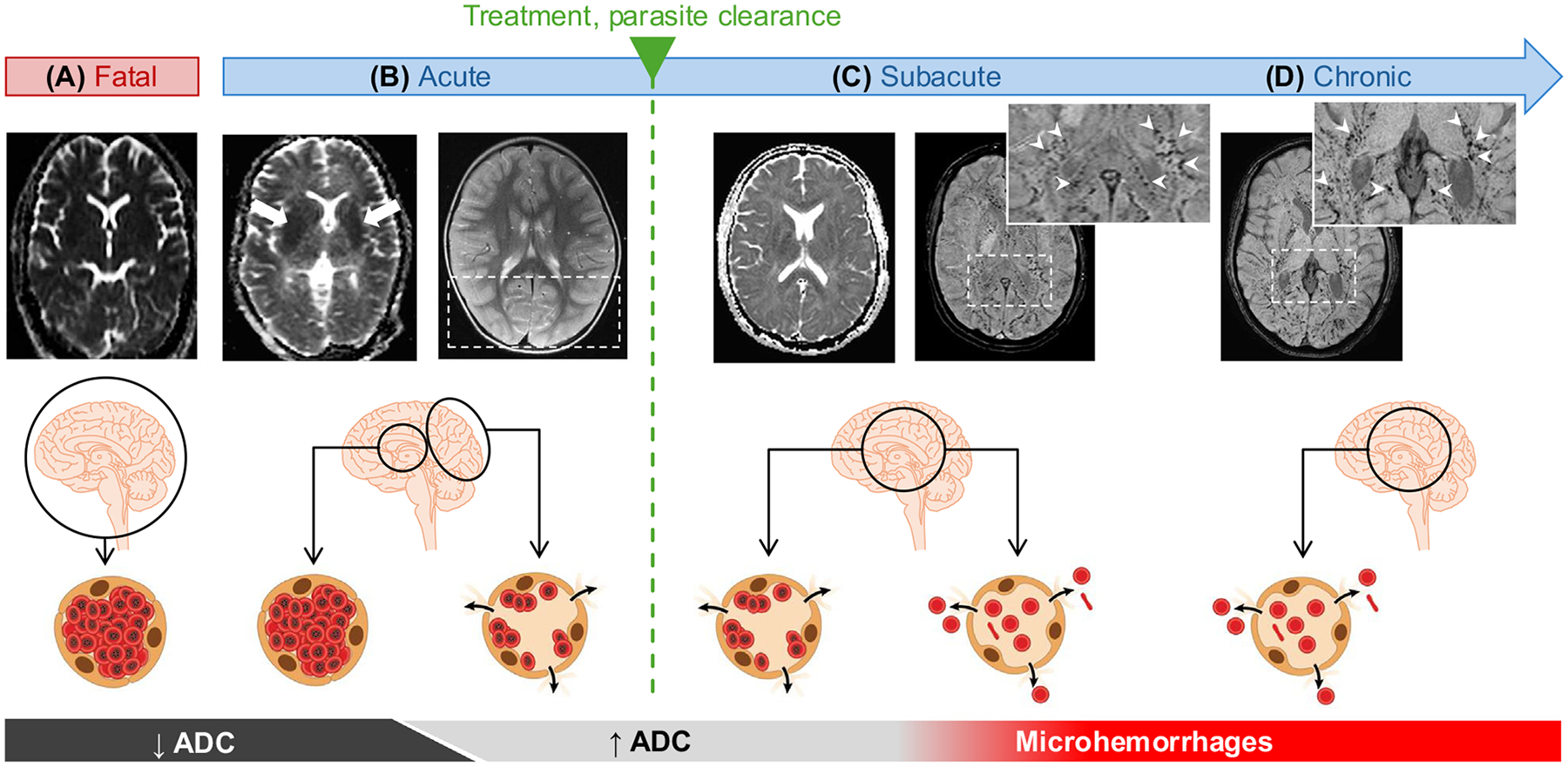
Proposed sequence of pathogenic events in fatal and nonfatal adult cerebral malaria (CM). Fatal CM in adults is associated with a profound and global hypoxic brain lesion resulting from *P. falciparum*–infected red blood cell (iRBC) sequestration and microvessel congestion (A). In survivors, CM upon admission is associated with a decrease in apparent diffusion coefficient (ADC) values in the basal ganglia and subcortical white matter (B; acute phase, white arrows), as well as a mild to moderate brain swelling in the posterior part of the brain (B; acute phase, dashed box), indicative of cytotoxic and vasogenic edema, respectively. Upon treatment and progressive clearance of iRBC, vessels fragilized by sequestration plugging are unable to sustain the blood flow pressure, leading to vasogenic edema and/or microhemorrhages during the following 48–72 h (C; subacute phase, dark spots indicated by white arrowheads, zoomed area highlighted by the dashed box). In adults, microhemorrhages can persists for months in the subcortical and deep white matter structures following CM (D; chronic phase, dark spots indicated by white arrowheads, zoomed representative area highlighted by the dashed box). The representative image of the chronic phase was acquired 50 days postadmission. Radiological images compiled from [18,19,23].

Fatal CM in adults is associated with a profound and global cytotoxic edema in the brain, likely resulting from a combination of high parasite biomass [35], degree of microvascular sequestration, and congestion in the cerebral microvasculature [36] (Figure 2A). Sequestration in patients with CM has been associated with specific variants of ***Plasmodium falciparum* erythrocyte protein 1 (PfEMP1)** that bind to the endothelial protein C receptor (EPCR) in brain microvessels [37]. This severe hypoxic injury may be aggravated by platelet accretion, clumping, rosetting, and bridging locally [10] and in fatal CM is corroborated by elevated plasma lipocalin-2 and miRNA150, two markers of hypoxia [19]. In nonfatal cases, cytotoxic edema develops first in hypoxia-sensitive regions during the acute stage of CM and is observed upon hospital admission, mainly affecting the basal ganglia and the subcortical white matter (Figure 2B). The occurrence of acute cytotoxic edema explains why adjunctive steroids, hypertonic saline, and mannitol were not effective when administered upon admission in adult patients with CM [38]. In areas of lesser sequestration and vascular fragility, mild to moderate vasogenic edema is caused by an increased permeability of the BBB resulting from the disruption of cytoprotective interactions between activated protein C and EPCR, because PfEMP-1 variants binding to EPCR have been associated with the development of both CM and brain swelling in Indian adults [39]. Following successful treatment with artemisinin derivatives, iRBCs are cleared rapidly in survivors [40], and cytotoxic edema reverses [19]. It is then followed by vasogenic edema in the subacute stages [23], as described in other forms of hypoxic brain injury with subsequent reperfusion. These short-term changes can be visualized in sequential MRI of admitted patients recovering from CM [19,23]. We postulate that in CM, the high volume of iRBCs sequestered through their engagement of PfEMP1 with EPCR during the initial phase of the disease initiates cytotoxic edema [37,39] and then leads to an increased permeability of the BBB, resulting in vasogenic edema when the blood flow resumes (Figure 2C). It is likely that the systemic and local production of cytokines, release of parasite toxins such as hemozoin, uric acid, and others, as well as involvement of platelets and CD8^+^ T cells all contribute to this BBB impairment [10], albeit to a lesser extent. In addition, the transient plugging of microvessels may also alter the function of BBB by creating local hypertension, which increases pressure on **tight junctions**. If increased local vascular pressure persists, tight junctions break, causing the BBB to breach and triggering microbleeds. We hypothesize that vasogenic edema is then followed by microhemorrhages, because damaged or previously thrombosed microvessels cannot withstand the pressure of normalized blood flow, leading to their rupture (Figure 2C). In both Indian and UK cohorts, microhemorrhages persisted over 50 days postdischarge in some patients, a chronic condition potentially enhanced by the long-term endothelial activation and dysfunction reported following CM [41] (Figure 2D).

### UM

Patients with UM have lower parasite biomass and subsequent intravascular sequestration levels (Figure 3A, Key figure). The mild vasogenic edema identified by MRI in most UM cases (Figure 3B) is unlikely to result from BBB impairment caused by EPCR-binding iRBC (Figure 3C), because these PfEMP1 variants have been specifically associated with the development of brain swelling and CM [39]. Instead, we hypothesize that several other pathogenic mechanisms leading to endothelial dysfunction and increased BBB permeability are involved. These have been reviewed elsewhere [10] and include local inflammation triggered by **pathogen-associated molecular patterns** and **damage-associated molecular patterns** (Figure 3C).

### Severe noncerebral malaria

Patients with **severe noncerebral malaria (SNCM)** exhibit a wide spectrum of clinical disease, corroborated by high fluctuations in sequestration and vascular engorgement in the brain [36] and neuroimaging changes (Figure 3A,B). The latter range from CM patterns with decreased ADC in the basal ganglia [34] to UM patterns, with increased ADC values and generalized mild vasogenic edema [19] (Figure 3B). Due to their admission at different time points of the disease trajectory, this clinical group encompasses pre-CM patients with lower ADC in the basal ganglia and decreased GCS scores but without obvious coma [34]. There, the high parasite biomass and sequestration lead to local hypoxia in the basal ganglia that quickly reverses upon treatment and parasite clearance. AKI is frequent in SNCM and likely to exacerbate these nascent hypoxic lesions [11], whereas liver dysfunction, another common falciparum malaria complication, has been hypothesized to aggravate BBB impairment and contribute to vasogenic edema [12] (Figure 3B).

## Neurocognitive impairment

The current dogma that only a low proportion of adult patients with CM experience neurological sequelae [8] stems from few studies using limited neurological assessments at the time of discharge (Table 1, highlighted rows), and large cohorts with long-term neuropsychometric follow-up are currently lacking [42]. Detailed neurocognitive assessments significantly increase the detection of sequelae in this age group [23], and the clinical consequences of MRI changes seen in patients with UM and SNCM have never been explored. We postulate that there are varied degrees of altered neurocognitive function following infection across the entire spectrum of falciparum malaria in adults. In CM, this is supported by studies using long-term follow-up and targeted assessments (Table 1). Patients with SNCM have increased plasma levels of S100B [34], a biomarker of brain injury associated with long-term changes in neurocognitive function in other pathologies [43–46]. In both CM and SNCM, the frequent development of AKI has been shown to result in impaired neurocognition in children, either directly or through subsequent chronic kidney disease, which both contribute to negative effects on cerebral function [11]. The same processes are highly likely to occur in adults, where AKI has been associated with increased cognitive impairment or dementia risk [47]. Hepatic dysfunction is another common complication in adult CM and SNCM, which has recently been hypothesized to cause malaria-induced neurocognitive and behavioral sequelae [12]. Indeed, a high proportion of adults with liver dysfunction develop neurological and neuropsychiatric syndromes [48]. Transient mild to moderate vasogenic edema identified by MRI in most patients with UM [19] may result in mild to moderate neurocognitive impairment, as seen in other conditions such as anemia [49].

## Retinopathies

The retina is a visible extension of the central nervous system, and its examination has significantly contributed to our understanding of pediatric CM pathogenesis. Malarial retinopathy is now widely employed in clinical care and is routinely integrated into clinical studies to refine inclusion criteria in children [50]. However, the strong association reported between retinal changes and pediatric CM is not clear in adults, where retinopathies are also seen in patients with UM and SNCM [18,19,51,52]. This may result from age-specific differences in CM pathophysiology [19]. A more likely explanation is that retinal changes also reflect pathogenic processes in the brain in adults with falciparum malaria, but their wider disease spectrum described above does not allow clear-cut CM diagnostics using retinopathies. This picture is further complicated by the high frequency of AKI in severe falciparum malaria, which can also trigger or aggravate retinal changes [53]. The presence of retinopathies in adults with nonfalciparum malaria could also indicate brain involvement, regardless of the presence of coma. Indeed, retinal changes have been described in teenagers and adult patients with vivax malaria [54] and in adults with knowlesi malaria [55]. AKI [56] and brain involvement can occur in both species [57,58], with unknown long-term impacts. It is also plausible that retinal changes are not sensitive and specific enough for CM diagnosis in adults, owing to the incidence of other systemic and retinal diseases, such as diabetes, hypertension, systemic lupus erythematosus, and sarcoidosis. Children may have ‘healthier’ retinas than adults, allowing a higher diagnostic sensitivity and specificity of retinopathies in pediatric CM.

Combined MRI, retinal, and neurocognitive assessments are needed to rigorously examine correlations between brain and eye changes in adult patients as well as sequelae, taking into account the effects of comorbidities and distant organ effects such as AKI or hepatic dysfunction. This will determine whether retinopathies could be used as a surrogate for brain lesions in adults with malaria, allowing their identification and potential patient neurorehabilitation in the future.

## Concluding remarks and future perspectives

There is increasing recognition that *Plasmodium falciparum* infection leads to pathological cerebral alterations identified by MRI and is associated with elevated circulating biomarkers of brain injury, regardless of coma. These changes are more pronounced in patients with CM but also occur in patients with severe non-CM and patients with UM, highlighting a continuum of disease with specific MRI patterns at each end of the spectrum. Nonfalciparum malaria can also impact brain function. CM caused by *Plasmodium vivax* is now widely recognized, and a study reported the long-term negative effects of acute vivax malaria on executive and cognitive functions in adults [59]. Similarly, neurocognitive impairment was recently observed up to 2 weeks postdischarge in an adult case of severe *Plasmodium knowlesi* infection with impaired consciousness [60]. Elevated plasma markers of brain injury and endothelial activation were reported in patients with severe and nonsevere knowlesi malaria, indicating that mild brain injury may be frequent [61].

Collectively, these findings underscore the urgent need for large and well-designed cohort studies with serial follow-up assessments to evaluate the effects of both falciparum and nonfalciparum malaria infection on neurocognition in adults (see Outstanding questions). As postulated elsewhere, the deficits may be subtle and undetected unless individuals undergo stringent cognitive-behavioral assessments but are likely to impact the quality of life in survivors across the clinical spectrum of malaria infection [62]. A key aspect to consider in future studies is the standard use of targeted evaluations focused on predicted patterns of neurocognitive impairment reported postmalaria infection [63]. Such assessment in adults should include deficits in attention, language, memory, executive functions, and visuospatial skills, which can be carried out using the Addenbrooke’s Cognitive Examination III tool [64,65]. In parallel, investigating the predictive value of retinopathies and relevant biomarkers of neurovascular unit damage and hypoxia on admission [66] should be prioritized in this age group. Once validated in several cohorts, these new tools could help in identifying individuals at risk of developing neurocognitive sequelae and support clinical management, as well as potential future targeted neurorehabilitation efforts.

## Figures and Tables

**Key figure Figure 3.**
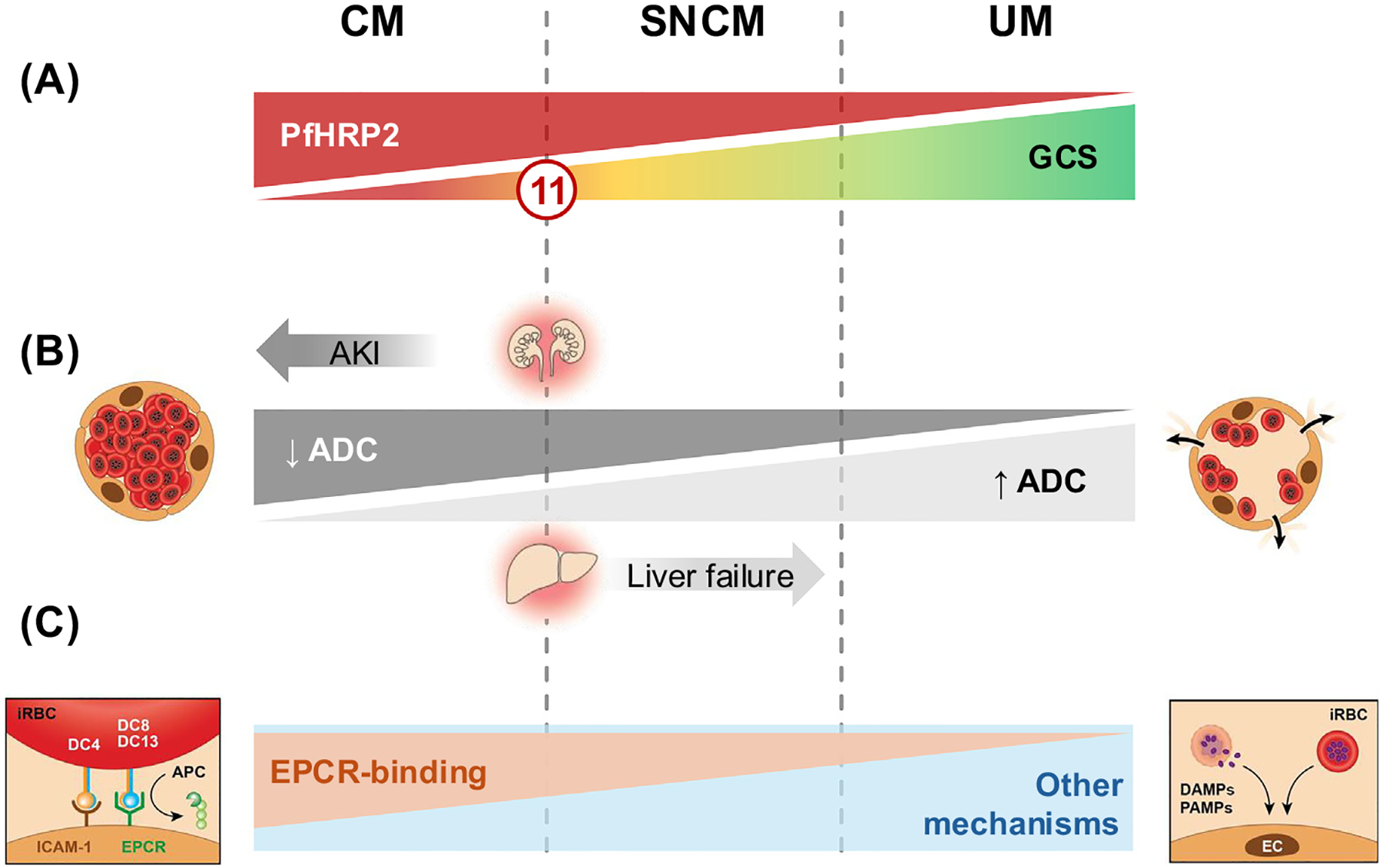
Summary of postulated acute pathogenic mechanisms in the brain across the disease spectrum of falciparum malaria in adults (A) Parasite biomass measured by circulating levels of *Plasmodium falciparum* histidine-rich protein (PfHRP2), Glasgow Coma Scale (GCS) score, and current World Health Organization cutoff for CM. (B) Main initial pathogenic events and associated neuroradiological signatures, postulated influence of acute kidney injury (AKI) and liver failure on apparent diffusion coefficient (ADC) values. (C) Factors influencing blood–brain barrier (BBB) permeability, including PfEMP1 interactions with endothelial protein C receptor (EPCR) (left panel), pathogen-associated molecular patterns (PAMPs), and damage-associated molecular patterns (DAMPs) released from rupturing *P. falciparum*–infected red blood cells (iRBCs), and receptor/ligand interactions between iRBCs and microvascular endothelial cells (EC; right panel).

**Table 1.** Summary of published neurologic and neurocognitive sequelae in adult CM and the assessment methods used

Country	Year	No. of subjects	Neurological examination	Neurocognitive ± psychometric assessments	Last postdischarge follow-up	Sequelae	Incidence^a^	Refs
Studies in endemic countries								
Angola	2013	22	Yes	Yes	2–6 months	Yes	-	[67]
**India**	**2002**	**441**	**Yes**	**No**	**15 days**	**Yes**	**10%**	[68]
**Bangladesh**	**1998**	**104**	**Yes**	**No**	**No follow-up**	**Yes**	**4%**	[69]
**Nigeria**	**1993**	**7**	**Yes**	**No**	**No follow-up**	**No**	**0%**	[70]
Studies in returning travelers								
UK	2023	2	Yes	Yes	>10 months	Yes	100%	[23]
Japan	2018	1	Yes	Yes	>10 months	Yes	100%	[31]
UK	2013	1	Yes	Yes	>20 months	Yes	100%	[71]
France	2001	6	Yes	Yes	>6 months	Yes	50%	[72]
Studies in war veterans								
USA	1997	40	Yes	Yes	>20 years	Yes	-	[73,74]

a When available.

## Outstanding questions

How frequent are neurocognitive sequelae in adults after *Plasmodium falciparum* infection? Do they also develop after mild acute clinical symptoms, including those caused by other malaria species?

Which biomarker or biomarker combination correlates best with the brain injury detected on MRI? Can retinopathies be used as an indicator of brain involvement in adults with falciparum malaria? In adults with nonfalciparum malaria?

Do brain changes persist over time besides microhemorrhages? Are these linked to decreased neurocognitive functions?

Which factors aggravate cerebral injury? Do AKI and liver failure contribute to neurocognitive decline in adults?

## Highlights

Recent neuroimaging studies using quantitative methods have allowed a better understanding of the pathogenic mechanisms underlying the development and progress of cerebral malaria in adults.

These methods have also uncovered brain changes in noncerebral malaria cases, including uncomplicated malaria, underlining a continuum of disease with specific magnetic resonance imaging patterns at each end of the spectrum.

Taken together, these findings highlight the potential risk for long-term effects on neurocognition never rigorously evaluated in large cohorts with longitudinal follow-up, using targeted evaluations to detect subtle impairment.

## Glossary

Apparent diffusion coefficient (ADC): quantitative measure derived from diffusion-weighted magnetic resonance imaging to determine the presence of cytotoxic and vasogenic edema
Cerebral malaria (CM): impaired consciousness due to *Plasmodium falciparum* infection defined by a Glasgow Coma Scale score below 11 in adults, with or without other complications
Damage-associated molecular patterns: in malaria infection, these are endogenous molecules released by host cells in response to *Plasmodium* infection, particularly during severe disease. These molecules signal cellular damage or stress and activate the immune system, contributing to both protective and pathological responses. They include free heme, host DNA, uric acid, and extracellular vesicles
Pathogen-associated molecular patterns: in malaria infection, these are conserved molecular structures of the *Plasmodium* parasite that are released upon infected red blood cell lysis and recognized by the host innate immune system. These patterns trigger immune responses and include glycosylphosphatidylinositol anchors, hemozoin, parasite histones, and parasite immunostimulatory nucleic acid motifs
*Plasmodium falciparum* erythrocyte membrane protein 1 (PfEMP1): family of highly variable antigenic proteins exported to the surface of *Plasmodium falciparum*–infected red blood cells, conferring them the ability to bind to endothelial receptors
Sequestration: process by which *Plasmodium falciparum*–infected red blood cells adhere to the endothelial lining of blood vessels in various organs. This phenomenon is a hallmark of severe falciparum malaria and contributes to its pathogenesis by obstructing blood flow, triggering inflammation, and causing tissue damage
Severe noncerebral malaria (SNCM): *Plasmodium falciparum* infection with significant and often overlapping complications or organ dysfunctions, including severe anemia, pulmonary complications, hypoglycemia, liver failure, and acute kidney injury (AKI)
Tight junctions: specialized connections between adjacent cells in epithelial and endothelial tissues that create a selective barrier, controlling the passage of molecules and ions between cells. They play a critical role in maintaining the integrity of tissue barriers, such as the blood–brain barrier and intestinal lining
Uncomplicated malaria (UM): *Plasmodium falciparum* infection leading to mild symptoms such as fever, moderate to severe shaking, chills, sweating, headache, nausea, vomiting, diarrhea, and anemia, with no indication of severe organ dysfunction
